# Multi-Omics analysis identifies a lncRNA-related prognostic signature to predict bladder cancer recurrence

**DOI:** 10.1080/21655979.2021.2000122

**Published:** 2021-11-30

**Authors:** Zhipeng Xu, Hui Chen, Jin Sun, Weipu Mao, Shuqiu Chen, Ming Chen

**Affiliations:** a Department of Urology, Affiliated Zhongda Hospital of Southeast University, Nanjing, China; b Department of Radiation Oncology, The First Affiliated Hospital of Nanjing Medical University, Nanjing, China; c Department of Urology, Xuyi People’s Hospital, Huaian, China; d Department of Urology, Zhongda Hospital Lishui Branch, Nanjing, China

**Keywords:** Multi-omics data, copy number variation, mutation, bladder cancer recurrence, long non-coding rnas^1^

## Abstract

Bladder cancer (BLCA) is one of the most common cancers worldwide with high recurrence rate. Hence, we intended to establish a recurrence-related long non-coding RNA (lncRNA) model of BLCA as a potential biomarker based on multi-omics analysis. Multi-omics data including copy number variation (CNV) data, mutation annotation files, RNA expression profiles and clinical data of The Cancer Genome Atlas (TCGA) BLCA cohort (303 cases) and GSE31684 (93 cases) were downloaded from public database. With multi-omics analysis, twenty lncRNAs were identified as the candidates related with BLCA recurrence, CNVs and mutations in training set. Ten-lncRNA signature were established using least absolute shrinkage and selection operation (LASSO) and Cox regression. Then, various survival analysis was used to assess the power of lncRNA model in predicting BLCA recurrence. The results showed that the recurrence-free survival time of high-risk group was significantly shorter than that of low-risk group in training and testing sets, and the predictive value of ten-lncRNA signature was robust and independent of other clinical variables. Gene Set Enrichment Analysis (GSEA) showed this signature were associated with immune disorders, indicating this signature may be involved in tumor immunology. After compared with the other reported lncRNA signatures, ten-lncRNA signature was validated as a superior prognostic model in predicting the recurrence of BLCA. The effectiveness of the model was also evaluated in bladder cancer samples via qRT-PCR. Thus, the novel ten-lncRNA signature, constructed based on multi-omics data, had robust prognostic power in predicting the recurrence of BLCA and potential clinical implications as biomarkers.

## 1. Introduction

Bladder cancer (BLCA) ranks tenth among cancers in incidence, with an estimated 549,000 new cases and 200,000 deaths worldwide in 2018 [1]. Transitional cell carcinomas account for over 90% in BLCA [2]. Approximately 70% of patients with transitional cell carcinomas were diagnosed as non-muscle-invasive bladder cancer (NMIBC), the others were diagnosed as muscle-invasive bladder cancer (MIBC) [2,3]. Despite the development in multimodal treatment, both NMIBC and MIBC have the high rates of recurrence [3–6]. NMIBC presents high risk of recurrence ranging from 31% to 78% at five years, but are generally not life threatening [2,7]. For MIBC, recurrence-free survival (RFS) and 5-year overall survival (OS) rates after radical cystectomy are 68.0% and 57.7%, respectively [5]. The high risk of recurrence and progression contributes to high mortality in MIBC [4,8,9]. Thus, it is clinically significant to screen for key biomarkers to assess the possibility of recurrence in patients with BLCA.

Long non-coding RNAs (lncRNAs), a major class of non-coding RNAs, are RNA transcripts with more than 200 base pairs [10]. Recent studies have also validated the roles of lncRNA in BLCA. Zhan et al. have reported that significantly upregulated expression of lncRNA *SOX2OT* was closely related with stemness phenotype in BLCA [11]. Wang et al. have verified that upregulation of *BLACAT2* made contributions to the BLCA lymphatic metastasis by upregulating the expression of VEGF-C in epigenetic mechanisms [12]. *LNMAT1* can promote lymphatic metastasis through epigenetically activating CCL2-dependent macrophage recruitment in BLCA [13]. These findings demonstrated that aberrant expression of lncRNAs was involved in BLCA initiation and progression, which represented their potential as diagnostic and prognostic biomarkers. Thus, many researchers have focused on predicting the prognosis of patients with BLCA basing on lncRNA profiles. For instance, Joep J et al. revealed that lncRNAs profiling could provide additional information for BLCA subtyping, which contributed to precision patient management [14]. Du et al. [15] constructed an epithelial-mesenchymal transition (EMT)-associated lncRNA signature to predict the prognosis of BLCA patients. Besides, Mao et al. [16] developed a ten-lncRNA signature to predict the outcome and immune status of BLCA. Additionally, Gao et al. [17] identified a six-lncRNA signature as a robust prognostic marker in BLCA through COX regression analysis. However, limited data on tumor etiology was taken into consideration when only transcriptome data was comprehensively analyzed with clinical features. Recently, some studies have paid attention to distinguish cancer patients with different clinical outcomes via multi-omics data [18–21]. Manikandan et al. [22] identified that amplified P4HA1 gene was related to hypoxia in breast cancer via multi-omics analysis. Zhao et al. [23] provided a novel insight into molecular subtypes for lung adenocarcinoma basing on multi-omics data (genomics, epigenomics, and transcriptomics). Chaudhary et al. [24] evaluated prognosis features of hepatocellular carcinoma patients via multi-omics data, which showed robustness in several external cohorts. However, multi-omics analysis has not been performed in constructing the lncRNA-associated prognostic model in patients with BLCA.

The previous studies have indicated the critical roles of lncRNAs in BLCA, and prompted the potential value of multi-omics analysis on constructing prognostic model. Hence, we assumed that multi-omics analysis may contribute to construct a robust lncRNA predictive model for BLCA recurrence, and intended to construct a novel lncRNA signature on the basis of multi-omics data, including transcriptome data, clinical data, copy number variation (CNV) data and mutation annotation data in the cohort of BLCA patients from The Cancer Genome Atlas (TCGA) database and GSE31684 dataset. We successfully constructed a ten-lncRNA signature for RFS of BLCA patients by a contemporary clinical-practical statistical method, least absolute shrinkage and selection operation (LASSO), and COX regression model. Our results validated that the ten-lncRNA signature could act as an independent prognostic predictor of BLCA recurrence. Besides, the novel lncRNA signature may be associated with tumor immunology. We present the following article in accordance with the TRIPOD reporting checklist.

## 2. Materials and methods

### 2.1. Pre-processing of lncRNA-associated information and clinical data from public databases

The RNA expression profiles, clinical data and genomic copy number variation data in the TCGA database were obtained via the UCSC cancer browser (https://xenabrowser.net/datapages/). And the mutation annotation file (MAF) was extracted via GDC client. After downloading the ‘fragments per kilobase of transcript per million fragments sequenced’ (FPKM) data of RNA-Seq from TCGA database, we obtained the lncRNA expression profiles by cross-referring to ensemble ID of lncRNAs from GENCODE project [25]. Then, ‘transcripts per million’ (TPM) values were calculated according to the FPKM values. Finally, the TPM values were normalized by Z-score.

In addition, we downloaded the clinical data and transcriptome profiles of Series GSE31684 from GEO database (https://www.ncbi.nlm.nih.gov/geo/) [26]. The microarray raw data were downloaded from GEO database. We obtained the lncRNA probes from the manufacturer’s website (http://www.affymetrix.com), and mapped the probe sequences to the human genome (hg19) without mismatch. Next, 5076 probes fell into lncRNAs through re-annotation. Eventually, the expression data was normalized via the quantile-normalization approach [27,28].

Herein, we concentrated on BLCA recurrence. Hence, we excluded the BLCA samples whose RFS time was unknown, not described or less than 30 days. We randomly selected three-quarters of samples from the BLCA cohort in TCGA database as the training dataset. The main reason for selecting three-quarters of samples is to include more samples in the training dataset in order to obtain more stable results. To avoid the selection bias, we performed the re-randomizations for 1000 times, and calculated the AUC distribution of each dataset, mainly ranging from 0.65 to 0.76 (Supplemental figure 1A). Eventually, 303 cases from TCGA database and 93 cases from Series GSE31684 in GEO database were included in this study. We randomly selected 227 cases from all 303 cases in the TCGA database as the training datasets (n = 227). The first testing datasets consisted of all 303 patients in the TCGA database. The second testing datasets were the 93 samples from Series GSE31684 in GEO database. We constructed the lncRNA-related prognosis model on the basis of the training dataset. The prognosis model was validated in the testing datasets. This study was conducted in accordance with the Declaration of Helsinki (as revised in 2013).

### 2.2. Gene CNV analysis

As for the copy number variation data obtained from TCGA database, the genomic regions with somatic copy number alterations (SCNA) were determined by GISTIC 2.0 [29]. The GISTIC peaks of amplification or deletion with p-value < 0.05 were regarded as significance. Next, in order to identify lncRNAs with CNV in BLCA, we mapped the genomic regions to GISTIC peaks.

### 2.3. Identification of lncRNAs associated with significantly mutated genes (SMGs)

Based on the MAF files from TCGA database, we identified SMGs with q-value < 0.05 using Mutsig 2.0 algorithms [30]. In order to identify the SMGs-related lncRNAs, we labeled the samples in the training datasets with the mutation or non-mutation of each SMG. Abnormal expression of lncRNAs associated with each SMG was determined by rank sum test with p-value < 0.01.

### 2.4. Identification of BLCA recurrence-associated lncRNAs

Univariate COX regression analysis was performed to screen out the lncRNAs associated with RFS. So as to further assess which lncRNA could act as the dependent variable factor, multivariable COX regression analysis was conducted. R software and bioconductor were utilized for analysis. P-value < 0.05 was considered as significant.

### 2.5. Construction of the lncRNA signature

On the basis of univariate COX regression analysis, gene CNV analysis and identification of SMGs-related lncRNAs, the lncRNAs associated with CNV, gene mutation and RFS time in BLCA were screened out as candidate genes for developing the lncRNA signature. Next, we intended to further select the candidate genes and construct a prognostic model with high accuracy by utilizing LASSO model [31]. The ‘glmnet’ package and R software were applied to perform LASSO regression algorithm.

After further selecting the candidate genes by LASSO regression algorithm, multivariable Cox regression analysis was performed on reserved candidate genes. Those lncRNAs with the lowest Akaike information criteria (AIC) value were screened out as the final candidate genes [32]. Next, the following formula was utilized to compute the risk score:

$$Risk\;Score\;=\overset{n}{\underset{i=1}{\sum }}\left(Coefficient_{lncRNAi}\;\times \;Expression_{lncRNAi}\right)$$

On the basis of training dataset, we performed ROC (receiver-operating characteristic) curves analysis with the AUC (area under curve) at five years of RFS. Next, the Youden’s index was calculated as the optimal cutoff point to distinguish BLCA cases into high or low recurrence risk sets [33]. Kaplan-Meier RFS curve analysis and the log‐rank test were applied in comparing RFS in two sets. The survft and survdif function of ‘survival’ packages and R software were applied to perform Kaplan-Meier RFS curve analysis and the log-rank test.

### 2.6. Validation of the prognosis power of lncRNA signature

We validated the signature in the validation datasets, namely the entire TCGA datasets and GSE31684 datasets. We utilized the Youden’s index to distinguish the cases in validation datasets into high and low recurrence risk sets. Next, we compare the RFS in high and low recurrence risk sets via Kaplan-Meier RFS analysis, log-rank test and further stratified analysis. In order to assess the prognostic power of lncRNA signature, we performed univariate and multivariate COX regression analysis in training dataset and testing datasets.

### 2.7. Functional enrichment analysis and tumor immune microenvironment characteristics

Gene Set Enrichment Analysis (GSEA) was performed based on the training dataset [34,35]. The RNA expression profiles were used as the input file and labeled with lncRNA signature-based risk score. The CIBERSORT method was performed to evaluate the relative abundance of immune cell profiling [36]. To evaluate the components in each sample, we applied the ESTIMATE algorithm to calculate the score of stromal, immune and ESTIMATE score in each sample [37]. In addition, the correlation was further analyzed with Spearman Rank correlation.

### 2.8. Clinical samples and cell lines

The 33 cases of BLCA specimens used in our study were collected from the Shanghai Tenth People’s Hospital of Tongji University, China. The 33 patients underwent radical cystectomy from May 2014 to June 2014. The 33 cases were staged based on the 7th AJCC staging system. This study was approved by the Ethics committee of the Shanghai Tenth People’s Hospital of Tongji University (SHSY-IEC-4.1/19-120/01). Prior informed consent was obtained from all of the patients. The bladder transitional cell carcinoma cell lines (UM-UC-3 and T24) were cultured in DMEM (Gibco, USA) containing 10% fetal bovine serum (FBS; Gibco, USA).

### 2.9. Transfection and qRT-PCR

In order to knockdown the expression of AGAP2-AS1, small interfering RNA (siRNA) was purchased from GenePharma (Shanghai, China). The siRNAs were transfected into cells using Lipofectamine 2000 (Invitrogen) according to the manufacturer’s instructions. We isolated the total RNA from cell lines and tissues using TRIzol (Takara, Japan) based on the manufacturer’s instructions. LncRNA reverse transcription were conducted with a New Poly(A) Tailing Kit (ThermoFisher Scientific) and PrimeScript RT Master Mix Kit (RR036A, TaKaRa), respectively. qRT-PCR was performed using a Universal SYBR Green Master Mix (4,913,914,001, Roche) with a 7500 Real-Time PCR System (Applied Biosystems, USA). We normalized the relative lncRNA expression levels to GAPDH, respectively. The sequences of siRNAs and primers in this study were listed in Supplemental table 6.

### 2.10. Cellular proliferation assay and transwell assay

For the CCK-8 assay (CCK-8, Dojindo, Kumamoto, Japan), we first seeded cells in triplicate in a 96-well plate at a density of 2000 cells per well. At the indicated time points, we added 10 μL CCK-8 solution to each well. After a 2 h incubation, the absorbance was determined using a microplate reader. For the colony formation assay, we seeded cells in six-well plates at a density of 500 cells per well and cultured the plate for 2 weeks. Subsequently, we fixed the cells in 75% ethanol and stained them with crystal violet. Colonies were observed and counted under a light microscope. Cell migration was analyzed using Transwell chambers (Corning, USA). Cells were cultured in serum-free DMEM in upper chamber to inhibit cell proliferation.

## 3. Results

In this study, we performed the comprehensive analysis of transcriptome data, clinical data, CNV data and mutation annotation data in the cohort of BLCA patients from TCGA database and GSE31684 dataset. We constructed a ten-lncRNA signature for RFS of BLCA patients via LASSO and Cox regression model. Next, our results validated that the ten-lncRNA signature could act as a robust and independent prognostic predictor of BLCA recurrence in the training and testing datasets. Further analysis indicated that the novel lncRNA signature may be associated with tumor immunology. In addition, we verified that ten-lncRNA signature constructed via multi-omics analysis had better performance in predicting BLCA recurrence than the two reported lncRNA signature models.

### 3.1. Screening of BLCA relapse-associated lncRNAs

We pre-processed a series of information from the public database, including extraction of the RNA expression profiles, array re-annotation, exclusion of the samples with unclear RFS state, and randomly assigning cases into the training or validation cohorts as mentioned in the Materials and Methods. Next, we overviewed the clinical data of the training dataset and testing datasets, and displayed relevant clinical information including age, relapse state, gender, TNM stage, subtype, grade and tumor stage for each dataset in supplemental table 1. Next, univariate Cox regression analysis was conducted to identify those lncRNAs related to RFS in training dataset. 38 lncRNAs were chosen as the candidates for the subsequent study, and the results of univariate Cox regression analysis were shown in table 1 and supplemental table 2.

### 3.2. Analysis of gene CNV in BLCA

**Figure 1.**
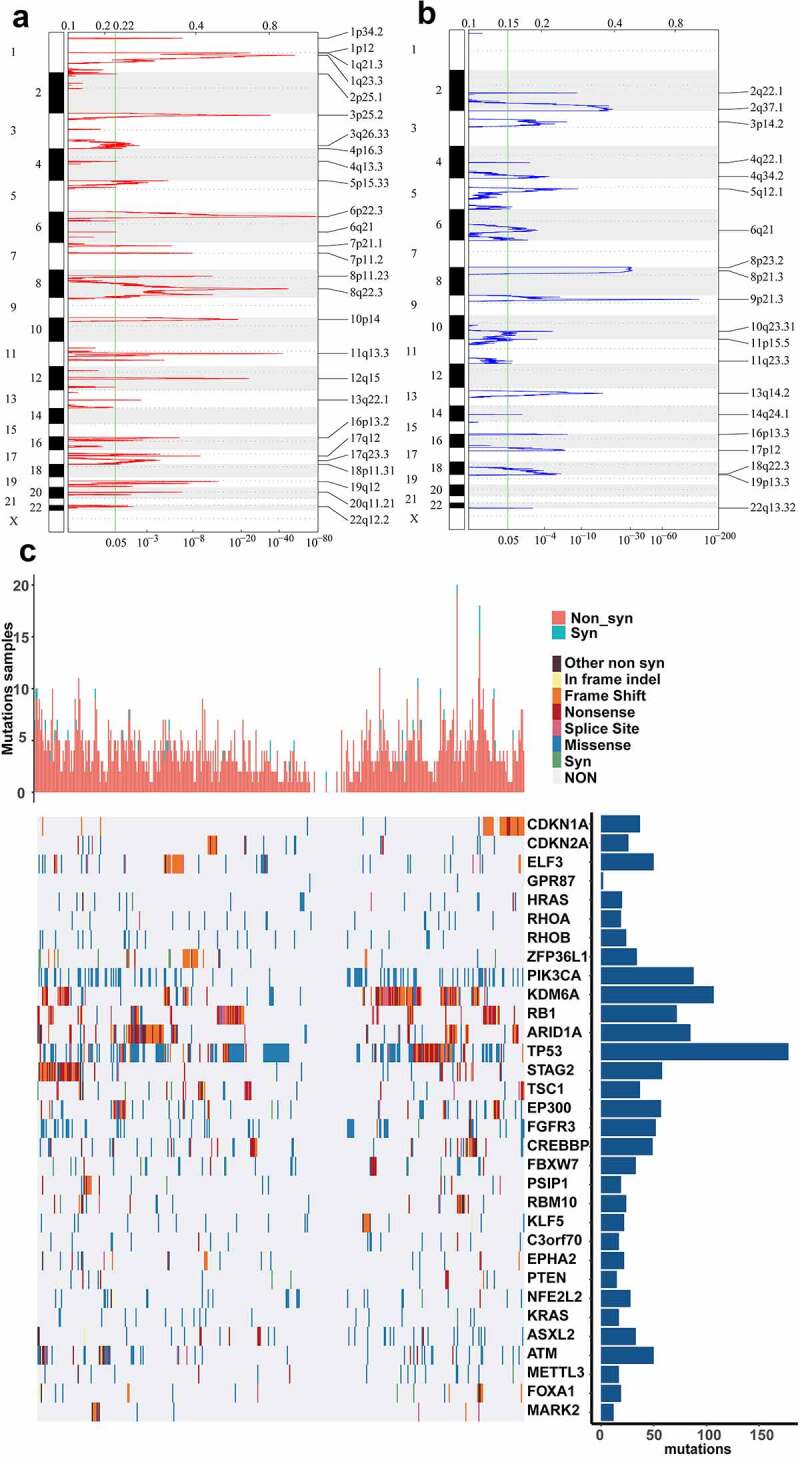
CNVs and mutations analysis of genome loci in BLCA. (a) The significantly amplified fragments (red) in BLCA genome were shown. (b) The significant deleted fragments (blue) in BLCA were shown. The rows are arranged according to the genome loci. (c) Distribution of mutations in 32 genes with significant mutation frequencies on basis of training dataset. Upper bar graph showed the total number of non-synonymous and synonymous mutations of 32 genes in each patient. And right histogram showed the number of clinical samples with mutations in each gene among the 32 genes

In past decades, more and more evidences demonstrated that genomic alterations could contribute to aberrant expression of lncRNA in various cancer [38–40]. Here, we identified the genes with significant genomic amplification or deletion in BLCA. Significant amplifications were shown in Figure 1(a). We documented genes with significant amplifications in supplemental table 3, such as significant amplification of *LINC00709* on segment 10p14 (q-value = 1.23e-19), significant amplification of *LOC101929622* on segment 8p11.23 (q-value = 2.05e-12) and significant amplification of *LINC01195* on segment 16p13.2 (q-value = 7.49e-07). Eventually, 717 genes were identified as amplified genes. The significant deletions in BLCA were shown in Figure 1(b). The genes significantly deleted on each fragment were recorded in supplemental table 4, such as significant deletion of *LOC100286922* on segment 2q37.1 (q-value = 3.05e-21), significant deletion of *LOC101929066* on segment 8p21.3 (q-value = 2.96e-32) and significant deletion of *LINC00208* on segment 8p23.2 (q-value = 3.28e-31). We identified 875 deleted genes in total.

### 3.3. Mining of lncRNAs associated with significantly mutated genes (SMGs)

We identified significant mutations according to TCGA mutation annotation data via Mutsig 2.0, and obtained 32 genes with significant mutations. Based on the TCGA training dataset, the distribution of synonymous mutations, missense mutations, frame insertions or deletions, frame movements, nonsense mutations, splice sites and other nonsynonymous mutations in the 32 genes were analyzed and shown (Figure 1(c)). The upper graph showed the amount of nonsynonymous and synonymous mutations of the 32 genes in every case. And the right histogram showed the number of clinical samples with mutations in each gene among the 32 genes. It has been reported that some of the 32 genes were closely associated with tumor initiation and progression, such as *CDKN1A, CDKN2A, ELF3, HRAS, PIK3CA, RB1* and so on. Then, we intended to identify the lncRNAs related to gene mutation in the 32 genes. We used the mutation state in each gene as a label and analyzed the difference between the expression of each lncRNA in the mutant and non-mutant sets via rank-sum test. lncRNAs with p-value < 0.01 were considered to be significantly associated with the mutation in some gene. As a result, we identified 2665 lncRNAs whose expression was related to gene mutation (supplemental table 5).

### 3.4. Establishment of the relapse-associated lncRNA signature

**Figure 2.**
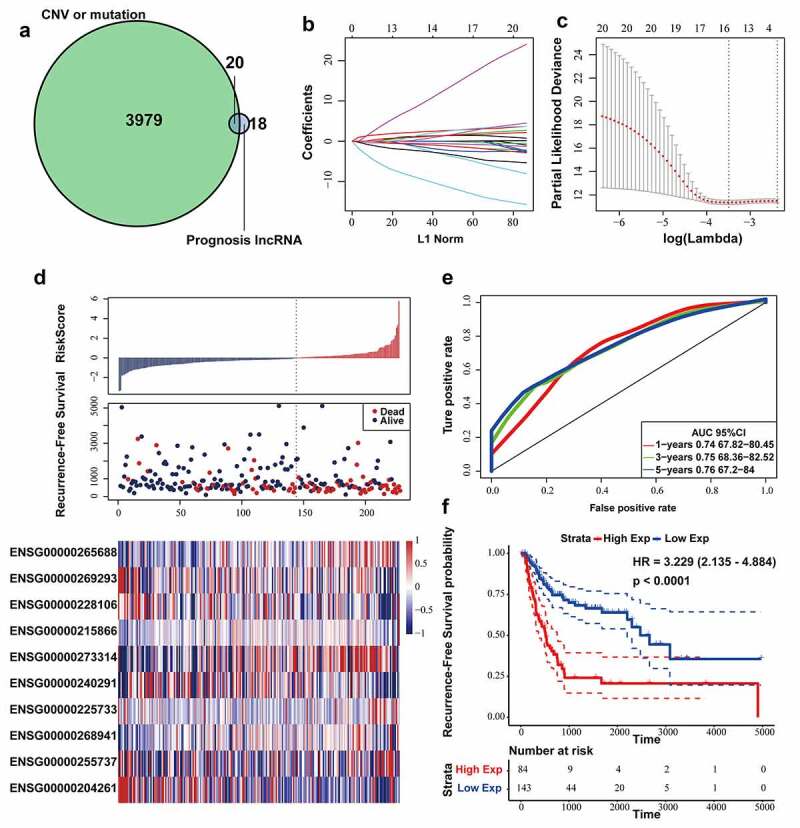
Construction and evaluation of the lncRNA signature based on multi-omics data. (a) Venn diagram about lncRNAs associated with genomic CNVs, gene mutation or BLCA recurrence. (b) The change trajectory of every independent variable. Horizontal axis represents the log value of independent variable lambda, and vertical axis represents the coefficient of independent variable. (c) Confidence intervals under each lambda. (d) Distribution of risk score, RFS and lncRNA expression of each case. (e) ROC curve analysis base on ten‐lncRNA signature. (f) Kaplan-Meier RFS curve analysis

According to above results, we found that 20 lncRNAs were related to genomic CNVs and gene mutation among 38 lncRNAs associated with recurrence (Figure 2(a)). In order to further select the candidate genes and construct a prognostic model while maintaining high accuracy, LASSO model was applied in developing a predictive signature with these screened 20 lncRNAs. LASSO method could build a penalty function to construct a refined model. LASSO evaluations of the coefficients of variables could effectively shrink coefficients and set some coefficients to zero. Hence, LASSO regression model retained the advantages of subset shrinkage and was an approach for biased estimation in processing the multi-collinearity data, which could select variables while estimating the model parameters and handle the multi-collinearity better. LASSO model performed the analysis of the change trajectory of each variable. More coefficients of independent variable approached zero with the lambda increasing gradually (Figure 2(b)). Then, we intended to construct the model with 3-fold cross-validation and analyzed the confidence interval under each lambda. We found that the model was optimal at lambda = 0.03046723 and selected the corresponding 16 lncRNAs as the candidate genes (Figure 2(c)).

Afterward, multivariate COX regression was performed on the 16 lncRNAs, and 10 lncRNAs with AIC: 841.77 (the lowest AIC value) were eventually selected as candidate genes whose details was displayed in Table 2. We computed risk score for each patient via the following formula:

$$\begin{array}{rl} & RiskScore=7.021*expLINC01711+5.153*\\ & \setminus expMAFG-DT-12.12*expZSCAN16-AS1\\ & +22.9*expAC005229.4+0.4837*expFGD5-\\ & AS1+4.255*expAGAP2-AS1+8.5*\\ & expLINC01356-9.161*expAL392172.1\\ & -10.86*expAL450384.2-5.485*expPSMB8-AS1 \end{array}$$

In the Cox regression analysis, *LINC01711, MAFG-DT, AC005229.4, FGD5-AS1, AGAP2-AS1* and *LINC01356* had positive coefficients, indicating that upregulation of these 6 lncRNAs was related to shorter RFS time. However, *ZSCAN16-AS1, AL392172.1, AL450384.2* and *PSMB8-AS1* with negative coefficients were considered as beneficial prognostic factors in BLCA.

The RFS status, risk score and expression of 10 lncRNAs in each patient from the training datasets were shown in Figure 2(d). According to the 5-year RFS prediction AUC in the training datasets, the Youden’s index (−0.4533178) was calculated as the optimal cutoff point to classify the samples into high (n = 84) or low (n = 143)-risk sets (Figure 2(e)), and Kaplan-Meier RFS analysis was performed (log-rank test p-value < 0.0001, HR = 3.229) (Figure 2(f)).

### 3.5. Validating prognostic power of the relapse-associated lncRNA signature

**Figure 3.**
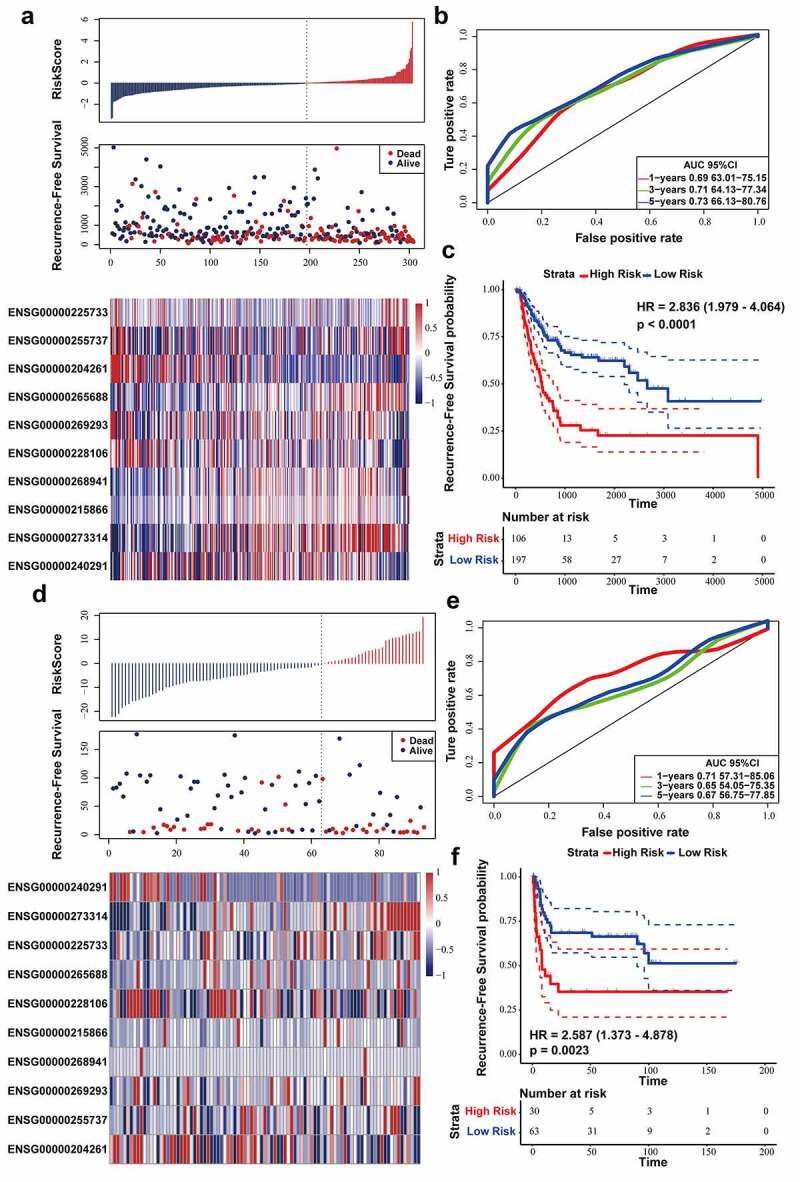
Evaluating the prognostic power of ten-lncRNA signature in the testing datasets. (a) Distribution of risk score, RFS and lncRNA expression of each case in the entire TCGA dataset. (b) ROC curve analysis of ten-lncRNA signature in the entire TCGA dataset. (c) Kaplan-Meier RFS curve analysis of high and low relapse‐risk sets in the entire TCGA dataset. (d) Distribution of risk score, RFS and lncRNA expression of every patient in GSE31684 dataset. (e) ROC curve analysis of ten-lncRNA signature in GSE31684 dataset. (f) Kaplan-Meier RFS curve of high and low relapse‐risk groups according to ten-lncRNA signature in GSE31684 dataset

Firstly, we verified ten-lncRNA signature in the entire TCGA dataset. We utilized the Youden’s index, calculated based on training dataset, to distinguish the cases in the entire TCGA datasets into high (n = 105) and low (n = 196)-risk sets. We analyzed RFS, risk score and 10 lncRNAs’ expression level of each case in the entire TCGA dataset (Figure 3(a)). AUC for ten-lncRNA signature was 0.73 at the RFS in the fifth year (Figure 3(b)). As shown in Figure 3(c), we compared RFS time of high and low-risk sets (log-rank test p-value < 0.0001, HR = 2.887).

Afterward, we used GSE31684 dataset as the other testing dataset. According to the same cutoff point (Youden’s index), the cases in GSE31684 dataset were divided into high (n = 30) and low (n = 63)-risk sets. We analyzed RFS, risk score and 10 lncRNAs’ expression level in the GSE31684 dataset (Figure 3(d)), and AUC for RFS in the first, third, and fifth year was 0.71, 0.65, and 0.67, respectively (Figure 3(e)). Kaplan‐Meier RFS curve was utilized to compare RFS of high and low-risk sets (log-rank p value = 0.0023, HR = 2.587) (Figure 3(f)). These results suggested that the patients with higher risk score had shorter RFS time and higher recurrence rates in the testing datasets.

### 3.6. Evaluating whether ten-lncRNA signature had robust prognostic power in BLCA

**Figure 4.**
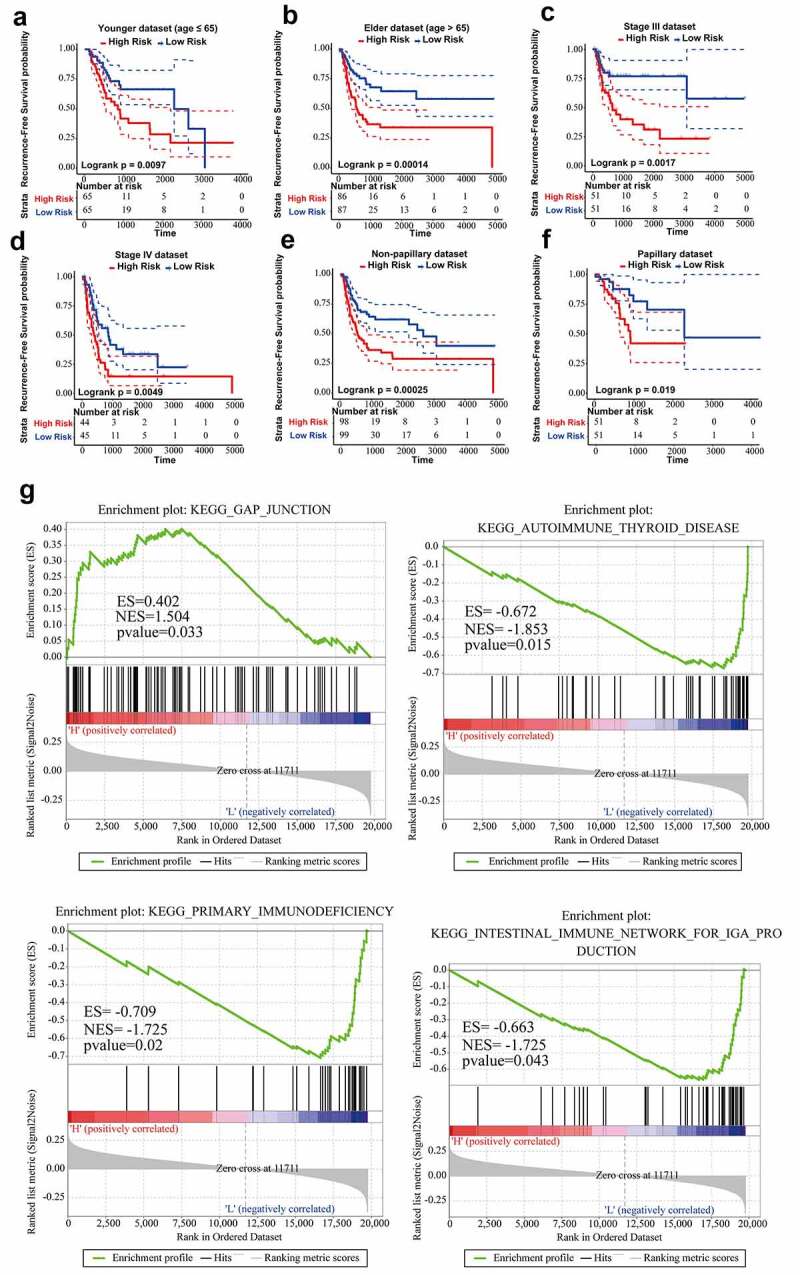
Stratified analysis on the basis of age, stage or subtype. (a,b) Kaplan-Meier RFS curve analysis in the younger or elderly dataset. (c,d) Kaplan-Meier RFS curve analysis in Stage III or Stage IV cohorts. (e,f) Kaplan-Meier RFS curve analysis in the non-papillary or papillary dataset. (g) Results of GSEA analysis in the TCGA training dataset

Firstly, stratified analysis was conducted to assess the relapse-predictive power of ten-lncRNA signature at different age, tumor stages or subtypes. All 303 cases in entire TCGA dataset were divided into younger (n = 130) and elderly (n = 173) datasets at the age (65 years old). As shown in Figures 4(a,b), ten-lncRNA signature could effectively distinguish each dataset into high and low relapse-risk sets. Next, all 303 cases were re-stratified into three different datasets according to the tumor stage (stage II, n = 108; stage III, n = 102; stage IV, n = 89). Ten-lncRNA signature could classify the tumor stage III or IV dataset into high and low relapse-risk sets via medium risk score (log-rank test p-value = 0.0017, Stage III; log-rank test p-value = 0.0049, Stage IV) (Figures 4(c,d)). However, ten-lncRNA signature could not distinguish these patients in stage II into different groups with different RFS (log-rank test p-value = 0.27, Stage II) (Supplemental Figure 1B). Finally, we stratified all 303 cases into non-papillary (n = 197) and papillary (n = 102) datasets based on subtypes. Ten-lncRNA signature could classify each dataset into high and low relapse-risk sets with different RFS (log-rank test p-value = 0.00025, Figure 4(e); log-rank test p-value = 0.019, Figure 4(f)).

To validate whether ten-lncRNA signature was an independent predictive factor for BLCA recurrence, we performed Cox regression analysis in the training and testing datasets. As shown in Table 3, we analyzed the association between RFS and clinical variables including ten-lncRNA signature.

In training dataset, univariate Cox regression analysis indicated that pathologic N stage, pathologic M stage, tumor stage and risk score were related to RFS of BLCA patients. And we found that risk score and pathologic N stage were related to RFS in the multivariate Cox regression analysis. In the entire TCGA dataset, univariate Cox regression analysis revealed that risk score, N stage, M stage and tumor stage were significantly associated with RFS of BLCA patients. And risk score and pathologic N stage were significantly associated with RFS in multivariate Cox regression analysis. In the GSE31684 dataset, we found that risk score was related to RFS in both univariate and multivariate Cox regression analysis. Taken together, ten-lncRNA signature has independent prognostic power for RFS prediction in patients with BLCA patients.

### 3.7. Pathway enrichment analysis and tumor immune microenvironment characteristics

In consideration of robust prognostic power of ten-lncRNA signature for BLCA recurrence, we supposed that these ten lncRNAs could take part in the progression of BLCA. We performed the Gene Set Enrichment Analysis (GSEA) among cohorts in the TCGA training datasets with the gene set named ‘c2.cp.kegg.v6.0.symbols’. The RNA expression profiles were used as input files and labeled with risk score of the ten-lncRNA signature. The pathways with significantly enrichment were shown in Table 4. As shown in Figure 4(g), GSEA revealed that some significantly enriched KEGG pathways were related to the tumorigenesis, tumor progression and immune disorders.

**Figure 5.**
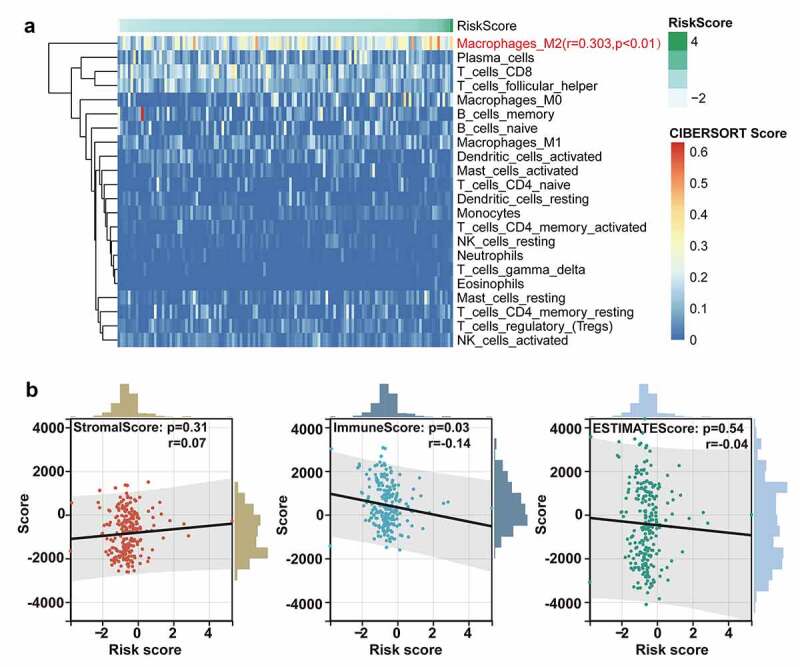
The analysis on the tumor immune microenvironment characteristics. (a) Evaluation of the correlation between the abundance of diverse immune cells and the ten-lncRNA signature via the CIBERSORT algorithm. (b) Evaluating the correlation between tumor microenvironment and the ten-lncRNA signature via the ESTIMATE algorithm

Considering the potential relationship between ten-lncRNA signature and immune disorders revealed in GSEA, we performed the analysis on the tumor microenvironment and the infiltration of immune cells. The CIBERSORT algorithm was performed to evaluate the abundance of diverse immune cells. As shown in Figure 5(a), the infiltration of M2 macrophage was positively correlated with the risk score based on the ten-lncRNA signature (R = 0.303, P-value < 0.01). Next, the association between tumor microenvironment and the ten-lncRNA signature was assessed via the ESTIMATE algorithm. The results indicated that the risk score had the significant and weak correlation with the immune score, which was consistent with the worse prognosis in the patients with higher risk score (Figure 5(b)).

### 3.8. Comparing ten-lncRNA signature with reported lncRNA signatures in BLCA

**Figure 6.**
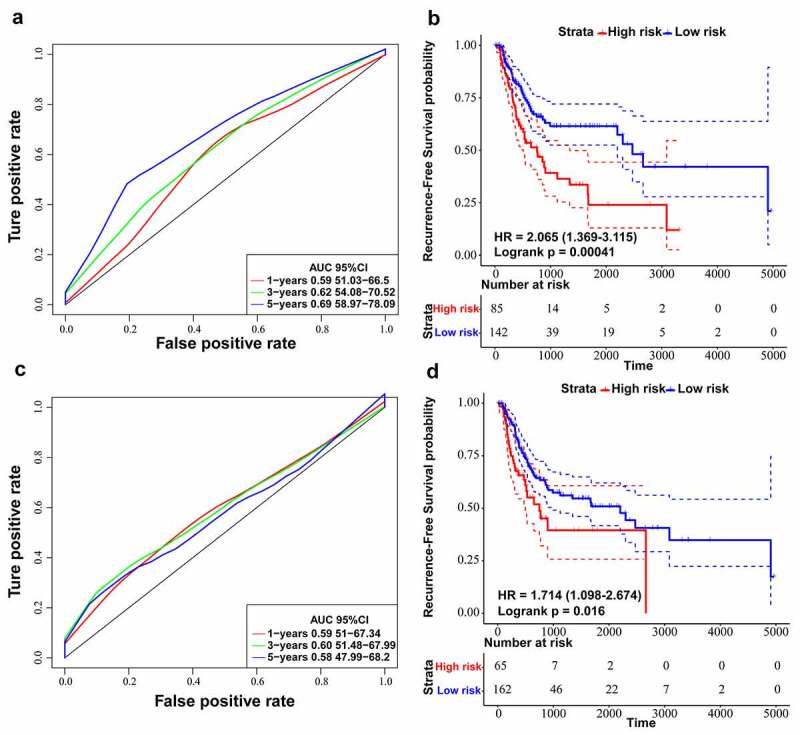
Comparing ten-lncRNA signature with two reported lncRNA signatures in BLCA. (a) ROC curve analysis and Kaplan-Meier RFS curve analysis on the reported 6-lncRNA signature in the TCGA training dataset. (b) ROC curve analysis and Kaplan-Meier RFS curve analysis on the reported 4-lncRNA signature in the TCGA training dataset

By searching for literature about lncRNA signatures, we chose two models associated with recurrence in BLCA: four-lncRNA signature (PMID: 28,060,759) [41] and six-lncRNA signature (PMID: 31,338,862) [17]. We recalculated risk scores of each patient in training dataset according to lncRNAs in the two selected models. Next, we utilized ROC curve analysis to classify cases into high and low-risk sets by Youden’s index. The results suggested that the AUC for RFS in the fifth year was 0.69 for the six-lncRNA signature (p-value = 0.00041) (Figure 6(a,b)) and the AUC for RFS in the third year was 0.60 for the four-lncRNA signature (p-value = 0.012) (Figure 6(c,d)). On the other hand, the AUC of 3-year and 5-year RFS prediction for the ten-lncRNA signature was 0.75 and 0.76, respectively (Figure 3(e)). By comparing the results of four-lncRNA signature, six-lncRNA signature and ten-lncRNA signature, we confirmed that the ten-lncRNA signature developed in this study had better performance in predicting BLCA recurrence.

### 3.9. Survival analysis of the ten-lncRNA signature in BLCA samples

**Figure 7.**
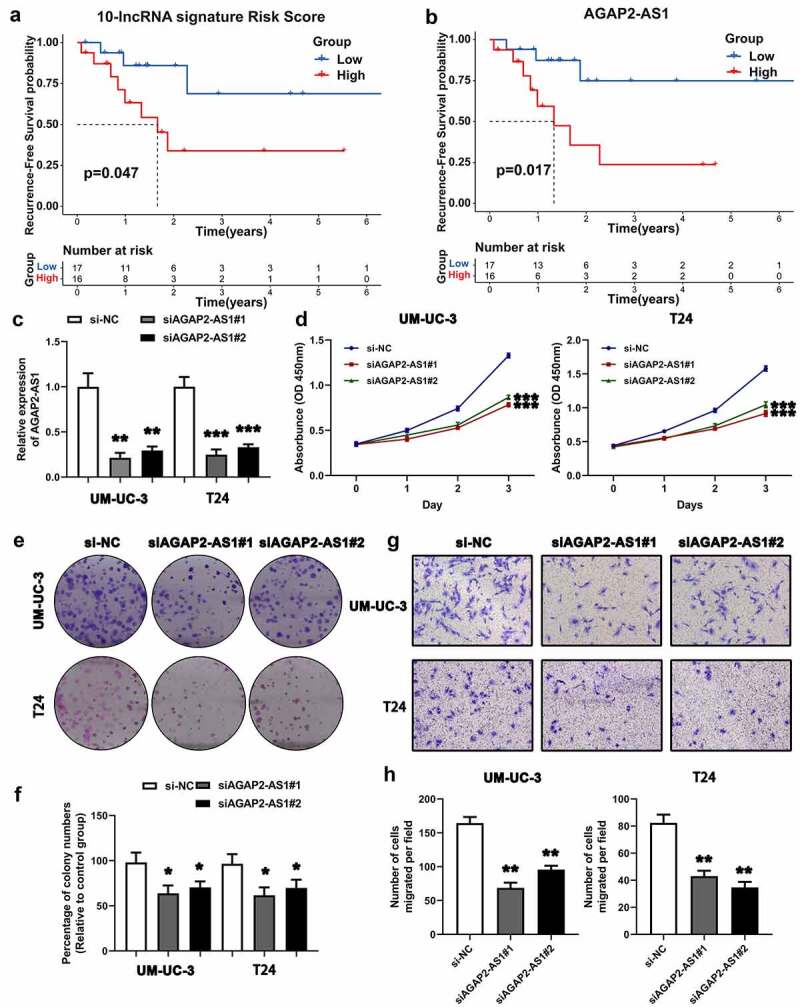
Survival analysis of the ten-lncRNA signature in BLCA samples and experimental study on the biofunction of AGAP2-AS1 on the BLCA cells. (a) Kaplan-Meier RFS curve analysis on the ten-lncRNA signature in the 33 BLCA samples. (b) Kaplan-Meier RFS curve analysis on the expression of AGAP2-AS1 in the 33 BLCA samples. (c) Evaluating the efficiency of AGAP2-AS1 knockdown via qRT-PCR. (d) CCK-8 assay on the effect of AGAP2-AS1 knockdown on cell proliferation. The OD value among different groups was found to be significantly different by two-way ANOVA. *p < 0.05; **p < 0.01; ***p < 0.001. The data are expressed as the mean ± SD. (e, f) Colony formation assay on the BLCA cells transfected with siRNA. (g, h) Transwell assay was used to evaluate the migration of BLCA cells transfected with siRNA

We determined the expression of the ten lncRNAs used to construct the relapse-associated lncRNA signature in the collected 33 BLCA samples. The clinicopathological characteristics of 33 patients was displayed in supplemental table 7. Based on the formula mentioned previously, we calculated the risk score for each sample. As shown in Figure 7(a), the survival analysis indicated that patients with higher risk score intended to have shorter RFS time, with marginal significance (P value = 0.047). In addition, we performed the survival analysis on the ten lncRNAs in the 33 samples. The results indicated that higher expression of AGAP2-AS1 and LINC01711 was significantly associated with higher possibility of BLCA recurrence (AGAP2-AS1, P value = 0.017; LINC01711, P value = 0.046) (Figure 7(b) and Supplemental figure 2).

### 3.10. AGAP2-AS1 knockdown suppresses cell proliferation and migration in BLCA cells

On the basis of survival analysis, we chose AGAP2-AS1 for further experimental study, because AGAP2-AS1 was most significantly associated with BLCA recurrence (P value = 0.017). LncRNA qRT-PCR was applied to evaluated the knockdown of AGAP2-AS1 (Figure 7(c)). The CCK-8 assay revealed that downregulation of AGAP2-AS1 expression presented a lower growth rate than the negative control in UM-UC-3 and T24 cells (Figure 7(d)). The colony formation assay further confirmed that AGAP2-AS1 knockdown could significantly inhibit BLCA cell proliferation (Figures 7(e,f)). On the other hand, the Transwell assay indicated that AGAP2-AS1 knockdown significantly inhibited cell migration compared with the control (Figures 7(g,h)). Results above suggested that AGAP2-AS1 knockdown could inhibit cell proliferation and migration in BLCA cells.

## 4. Discussion

Due to the development of high throughput RNA sequencing, expression pattern of lncRNAs was uncovered in diverse cancers [42,43]. Many studies have documented that expression pattern of some lncRNAs were specific in some cancer, even in different stage of some tumor [44–50]. In the recent years, a great number of studies have highlighted that dysregulation of lncRNAs was involved in cancer progression [51–54]. These evidences indicated that lncRNAs have the potential to act as biomarkers for prognostic prediction in human cancers. Hence, it is necessary to identify aberrant expression pattern of lncRNAs and reveal their possible roles in BLCA development and recurrence.

Herein, we performed multi-omics analysis of transcriptome, genomic CNV, mutation annotation and clinical data of BLCA in TCGA database, in order to find the lncRNAs whose aberrant expression was associated with BLCA recurrence. Our study uncovered that 38 lncRNAs were significantly related to BLCA recurrence through univariate Cox regression analysis. Then, CNV analysis revealed that 1592 genes had significant amplification or deletion in their genome loci and some genomic alterations contributed to the dysregulation of lncRNAs expression in BLCA. In addition, gene mutations analysis showed that there were a total of 32 genes with significant mutation, including some genes closely related to tumor initiation and progression. Further analysis indicated that expression pattern of 2665 lncRNAs was associated with these genes’ mutations. On the basis of our analysis mentioned above, we found that 20 lncRNAs were associated with gene mutation and CNV among 38 lncRNAs associated with BLCA recurrence. We further selected the 16 lncRNAs as candidates from the 20 lncRNAs using the LASSO model. Multivariate COX regression analysis eventually selected out 10 lncRNAs to develop a recurrence-associated lncRNA signature in BLCA.

Moreover, validation of the recurrence-associated lncRNA signature indicated that ten-lncRNA signature had predictive power for the recurrence of BLCA. We conducted Cox regression analysis in training dataset and testing datasets, and the analysis verified that ten-lncRNA signature was an independent prognostic factor for BLCA recurrence. Stratified analysis indicated that ten-lncRNA signature could effectively classify cases into high and low recurrence-risk sets in different subgroups. Taken together, ten-lncRNA signature based on multi-omics analysis could act as a robust and independent predictor for BLCA recurrence.

Furthermore, we performed the GSEA to reveal the potential pathways related to ten-lncRNA signature. The results of GSEA contained several significantly enriched BLCA-associated pathways, such as ‘gap junction’, ‘primary immunodeficiency’, ‘intestinal immune network for IgA production’, ‘autoimmune thyroid disease’ and ‘tight junction’. We supposed that ten-lncRNA signature may contribute to tumor immunity whose dysregulations could play a critical role in BLCA relapse.

By comparing ten-lncRNA signature with the reported four-lncRNA signature and six-lncRNA signature, we validated that ten-lncRNA signature had better performance in predicting BLCA recurrence than the two reported lncRNA signature models, which suggested that our approach of multi-omics analysis on transcriptome data, genomic CNV data, mutation annotation data and clinical data may be superior in constructing the prognostic signature.

Above all, the ten-lncRNA signature had robust predictive power, which was an independent prognostic factor for BLCA relapse. Hence, ten-lncRNA signature could have potential implications as prognostic markers for BLCA recurrence. On the other hand, the approach we utilized for developing biomarkers may contribute to studying cancer-associated RNA expression profiles in the future. Due to the limited samples collected from patients, the survival analysis based on the 33 BLCA patients revealed that only AGAP2-AS1 among the ten lncRNAs was associated with RFS in BLCA. Herein, we chose AGAP2-AS1 for further functional experiments, and the results revealed that AGAP2-AS1 knockdown could inhibit the cell proliferation and migration in BLCA cells for the first time. However, further investigation should be performed to validate biological functions and potential mechanisms of ten lncRNAs in BLCA.

## 5. Conclusion

The novel ten-lncRNA signature, constructed based on multi-omics data, had robust prognostic power in predicting the recurrence of BLCA and potential clinical implications as biomarkers for personalized management of BLCA.

## Supplementary Material

Supplemental Material

## Data Availability

Original data were uploaded to a recognized data repository (URL: https://zenodo.org/record/5153530#.YZtEZU5ByUk; DOI: 10.5281/zenodo.5153530)

## Research highlights

Multi-omics analysis on CNV, mutation annotation, RNA expression and clinical data.

Constructing a novel and robust lncRNA signature to predict BLCA recurrence.

The lncRNA signature may be associated with tumor immunology.

AGAP2-AS1 knockdown could inhibit cell proliferation and migration in BLCA cells.

## Author statement

Zhipeng Xu: Conceptualization, Methodology, Software, Writing - original draft preparation. Hui Chen: Data curation, Formal analysis, Writing - original draft preparation. Jin Sun: Validation, Writing - review and editing. Weipu Mao: Software, Visualization. Shuqiu Chen: Funding acquisition, Supervision. Ming Chen: Conceptualization, Funding acquisition, Supervision.
