# Supplementary material for: Multi-Omics analysis identifies a lncRNA-related prognostic signature to predict bladder cancer recurrence
Source: Bioengineered. 2021 Nov 30;12(2):11108–25. doi: 10.1080/21655979.2021.2000122 (PMC8810060; doi:10.1080/21655979.2021.2000122)
Supplement: Supplemental Material [file KBIE_A_2000122_SM6276.zip › supplementary/Supplemental table 1.docx]

**Supplemental Table 1. Clinical characteristics in the TCGA training datasets, the entire TCGA datasets and GSE31684 datasets**

| **Characteristic** | | **TCGA training datasets(n=227)** | **entire TCGA datasets(n=303)** | **GSE31684**  **(n=93)** |
| --- | --- | --- | --- | --- |
| **Age(years)** | <=65 | 98 | 130 | 29 |
|  | >65 | 129 | 173 | 64 |
| **Survival Status** | Non-Recurrence | 134 | 182 | 54 |
|  | Recurrence | 93 | 121 | 39 |
| **Gender** | female | 56 | 78 | 25 |
|  | male | 171 | 225 | 68 |
| **pathologic_T** | T 1 | 0 | 3 | 10 |
|  | T 2 | 73 | 94 | 17 |
|  | T 3 | 105 | 142 | 42 |
|  | T 4 | 26 | 35 | 19 |
| **pathologic_N** | N 0 | 136 | 185 | -- |
|  | N 1 | 26 | 36 | -- |
|  | N 2 | 37 | 45 | -- |
|  | N 3 | 5 | 6 | -- |
| **pathologic_M** | M 0 | 116 | 157 | -- |
|  | M 1/ M X | 110 | 145 | -- |
| **Tumor Stage** | Stage Ⅰ | 0 | 2 | -- |
|  | Stage Ⅱ | 81 | 108 | -- |
|  | Stage Ⅲ | 74 | 102 | -- |
|  | Stage Ⅳ | 70 | 89 | -- |
| **Subtype** | Non-Papillary | 153 | 197 | -- |
|  | Papillary | 72 | 102 | -- |
| **Grade** | High grade | 212 | 282 | 87 |
|  | Low grade | 12 | 18 | 6 |
