# Supplementary material for: Multi-Omics analysis identifies a lncRNA-related prognostic signature to predict bladder cancer recurrence
Source: Bioengineered. 2021 Nov 30;12(2):11108–25. doi: 10.1080/21655979.2021.2000122 (PMC8810060; doi:10.1080/21655979.2021.2000122)
Supplement: Supplemental Material [file KBIE_A_2000122_SM6276.zip › supplementary/Supplemental table 6.docx]

**Supplemental Table 6. The sequences of siRNAs and primers used in this study**

| **Definition** | **Sequences** |
| --- | --- |
| **Primers** |  |
| LINC01711 | F: 5’-CAAACAAACTGACAAATC-3’ |
|  | R: 5’-AAGAAAGTGAGAAGAGAG-3’ |
| MAFG-DT | F: 5’-TGTCTCAAGCCTATAATC-3’ |
|  | R: 5’-TCTCTATGTTACCAATGC-3’ |
| ZSCAN16-AS1 | F: 5’-TCTGACAATATGATACTAAGC-3’ |
|  | R: 5’-AATCTCAATGGTGGAATG-3’ |
| AC005229.4 | F: 5’-ATGGTAAGGTTGGAAGTA-3’ |
|  | R: 5’-AATGGTAAGATTGGAGAAG-3’ |
| FGD5-AS1 | F: 5’-GTGAACTTGGAGGAACTG-3’ |
|  | R: 5’-ACTTAGCATATCTACAACTACTG-3’ |
| AGAP2-AS1 | F: 5’-TCCTCCACTCCACCTCAA-3’ |
|  | R: 5’-GAGTCAGATCCTAGCCAGTC-3’ |
| LINC01356 | F: 5’-TGCCTAACAGAGAACAAT-3’ |
|  | R: 5’-AGTCAGTGAATGGAGATAG-3’ |
| AL392172.1 | F: 5’-GAGAGCAGACACCATCTTA-3’ |
|  | R: 5’-AACTTCAGGCAGGACTAG-3’ |
| AL450384.2 | F: 5’-TTACAACTACACGAGAAG-3’ |
|  | R: 5’-GCACAGAATAAGAGAAGA-3’ |
| PSMB8-AS1 | F: 5’-CCTATAATCCTAGCACTT-3’ |
|  | R: 5’-ACCATCCTCATAATATCG-3’ |
| **siRNA** |  |
| siAGAP2-AS1#1 | 5’-CUCCUCUCACACGACUUCGGTT-3’ |
| siAGAP2-AS1#2 | 5’- UGCUCUCCUCUCACACGACUTT-3’ |
| si-NC | 5’-UUCUCCGAACGUGUCACGUTT-3’ |
