# Supplementary figures and images for: Multi-Omics analysis identifies a lncRNA-related prognostic signature to predict bladder cancer recurrence
Source: Bioengineered. 2021 Nov 30;12(2):11108–25. doi: 10.1080/21655979.2021.2000122 (PMC8810060; doi:10.1080/21655979.2021.2000122)

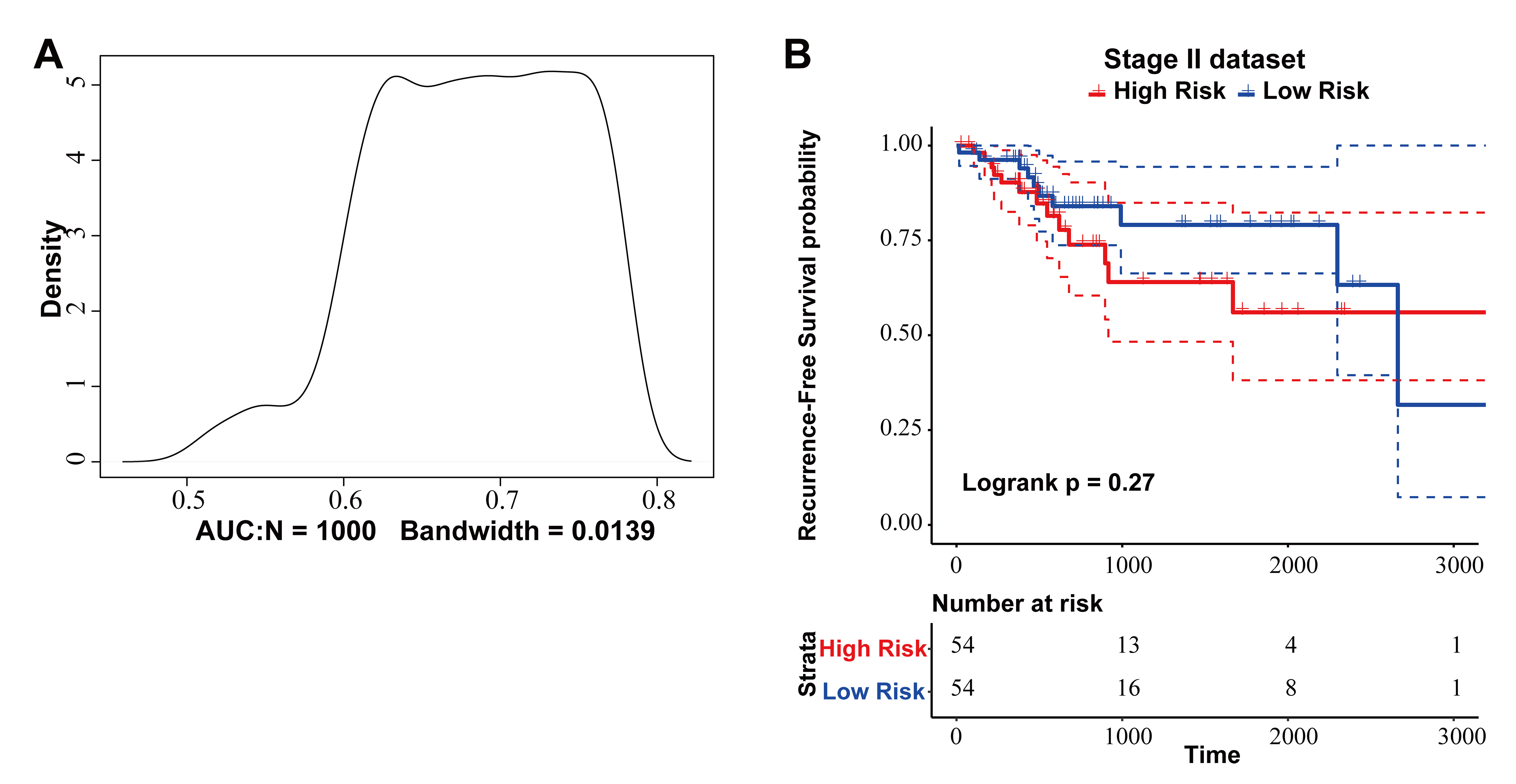

Supplement: Supplemental Material [file KBIE_A_2000122_SM6276.zip › supplementary/Supplemental figure1.tif]
